# Functionalized MoO_3_ Nanosheets for High‐Efficiency RhB Removal

**DOI:** 10.1002/gch2.202200154

**Published:** 2022-12-07

**Authors:** Yuxi Ma, Lifeng Wang, Dan Liu, Yuchen Liu, Guoliang Yang, Yijun Qian, Weiwei Lei

**Affiliations:** ^1^ Institute for Frontier Materials Deakin University Locked Bag 20000 Geelong Victoria 3220 Australia

**Keywords:** adsorption, ball milling, F‐MoO _3_ nanosheets, water purification

## Abstract

2D nanostructured materials have been applied for water purification in the past decades due to their excellent separation and adsorption performance. However, the functional 2D nanostructured molybdenum trioxide (MoO_3_)has rarely been reported for the removal of dyes. Here, functionalized MoO_3_ (F‐MoO_3_) nanosheets are successfully fabricated with a high specific surface area (106 cc g^−1^) by a one‐step mechanochemical exfoliation method as a highly effective adsorbent for removing dyes from water. According to the Raman, X‐ray photoelectron spectroscopy, Fourier transform infrared (FTIR), and selected area electron diffraction analysis, functional groups (hdroxyl groups, amide groups, amine groups and amino groups) are identified in the as‐prepared F‐MoO_3_ nanosheets. The attached functional groups not only facilitate the dispersal ability of F‐MoO_3_ nanosheets but also enhance the adsorption capacities. Thus, the performance (up to 556 mg g^−1^ when the initial concentration of Rhodamine B solution is 100 mg L^−1^) of as‐prepared F‐MoO_3_ nanosheets is almost two times higher than other reported MoO_3_ materials. Furthermore, the FTIR spectra, isotherm, and several factors (e.g., adsorbent dosage and adsorbate dosage) are also systematically investigated to explore the adsorption mechanism. Therefore, this work demonstrates that the F‐MoO_3_ nanosheets are a promising candidate for wastewater treatment.

## Graphical abstract

Functional molybdenum trioxide (F‐MoO_3_) nanosheets with a specific surface area of 106 cc g^−1^ are successfully fabricated by a one‐step mechanochemical exfoliation method. The adsorption capacity of the F‐MoO_3_ nanosheets is up to 556 mg g^−1^ for removing Rhodamine B dye from water, demonstrating great potential in industrial dye removal.

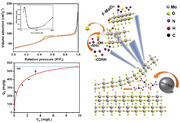

## 1 Introduction

In the past decades, various kinds of dyes have been widely used in different industries, such as textile, paper, cosmetics, and printing.^[^ ^1^, ^2^, ^3^ ^]^ Most dyes are considered as primary water contaminants due to their toxicity to the environment and human health.^[^ ^4^, ^5^ ^]^ Therefore, considerable attention was drawn to the effective removal of dyes from wastewater. To date, various methods, including sorption, filtration, degradation, and flocculation, are used to separate dyes from wastewater.^[^ ^6^, ^7^ ^]^ Among these methods, sorption is employed as an efficient way to purify water pollution in terms of flexible design, ease of separation, and low cost.^[^ ^7^, ^8^, ^9^ ^]^

The sorption capacities of adsorbents can be affected by several factors, including solution pH, contact time, reaction temperature, ionic strength, the volume of adsorbents, and the concentration of adsorbate solution.^[^ ^2^, ^4^, ^10^, ^11^, ^12^ ^]^ Among them, ionic strength is one of the most important and complicated factors.^[^ ^2^ ^]^ For the adsorption mechanism, dyes are mainly attracted by electrostatic attractions, hydrophobic interactions, surface functional group interactions, and hydrogen bonding interactions in aqueous solutions.^[^ ^13^, ^14^, ^15^, ^16^, ^17^ ^]^ The functional groups are proven to affect ionic strength, surface charge, and hydrophilicity.^[^ ^14^ ^]^ However, to date, the adsorption efficiency of dyes affected by the functional groups and molecular structure is rarely reported. Therefore, the relationship between functional groups on the surface of adsorbents and their adsorption performance still needs to be further explored.

In recent years, many adsorbents including activated carbon, resin, boron nitride, and polymers have been studied for dye removal from wastewater.^[^ ^18^, ^19^, ^20^, ^21^, ^22^, ^23^, ^24^, ^25^, ^26^, ^27^, ^28^ ^]^ However, these adsorbents still have a range of limitations, such as low adsorption capacity, poor recyclability, and potential secondary pollution.^[^ ^29^, ^30^ ^]^ Therefore, 2D materials are attracting great interest due to their unique physical and chemical features.^[^ ^31^, ^32^, ^33^, ^34^, ^35^, ^36^ ^]^ Molybdenum trioxide (MoO_3_) is a typical 2D material, which is structurally similar to hexagonal boron nitride and graphene, holding stacked layers together by weak van der Waals forces.^[^ ^37^, ^38^ ^]^ Molybdenum oxide is widely applied in many fields including supercapacitors, batteries, catalysts, and sensors, due to its unique structure and properties.^[^ ^39^, ^40^ ^]^ Recently, α‐MoO_3_ was proven as a potential effective adsorbent.^[^ ^41^ ^]^ Nevertheless, few investigations about dye sorption capacities and the mechanism of functional molybdenum oxide nanosheets have been reported up to date.^[^ ^42^ ^]^

Here, ball milling, as a one‐step exfoliation and functional method, was applied for developing nanostructured MoO_3_ in this work. Meanwhile, functional groups (hydroxyl (OH) group, amide (CONH) group, amine (NH_2_) group and amino (NH) group) are designed to attach on the surface of MoO_3_ for boosting the dye sorption ability of the functionalized MoO_3_ (F‐MoO_3_) nanosheets. The F‐MoO_3_ nanosheets exhibited superb performance in Rhodamine B (RhB) removal from water (*Q*_m_ = 556 mg g^−1^), which is much higher than the current reported literature, showing a bright future for wastewater treatments.

## 2 Results and Discussion

### 2.1 Synthesis and Characterization of F‐MoO_3_ Nanosheets

**Figure 1:**
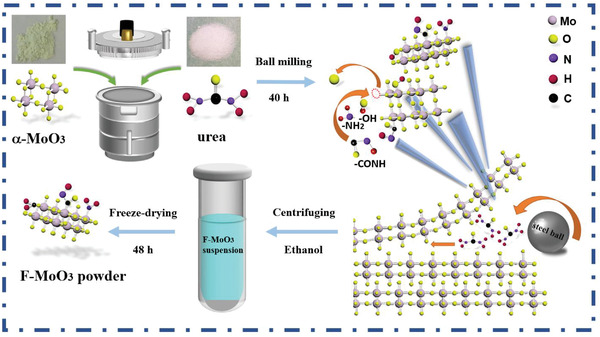
Schematic illustration of the preparation of the F‐MoO_3_ nanosheets.

As **Figure** **1** illustrates, the F‐MoO_3_ nanosheets were prepared via a one‐step method of the functionalization and exfoliation of commercial MoO_3_ with urea assistance. The usage of urea was not only for facilitating the exfoliation process but also for being a functional group source.

**Figure 2:**
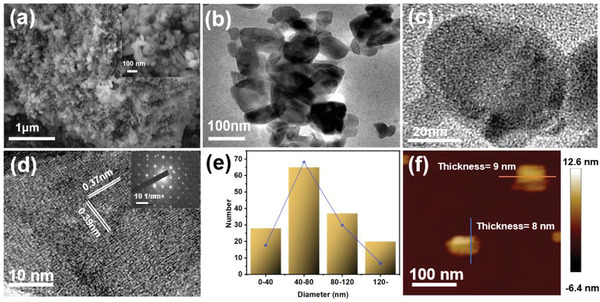
Morphology and structure characterizations of F‐MoO_3_ nanosheets. a) SEM image and enlarged SEM (inset) image of F‐MoO_3_ nanosheets. b,c) TEM image. d) HRTEM image, inset is SAED pattern. e) Lateral size distribution histogram of as‐prepared F‐MoO_3_ nanosheets. f) AFM image.

The surface morphology and microstructure of as‐prepared F‐MoO_3_ nanosheets were analyzed by scanning electron microscopy (SEM) (Zeiss Supra 55 VP) and transmission electron microscopy (TEM) (JEOL 2100) results. The SEM image of F‐MoO_3_ powder obtained by the freeze‐drying process and the TEM image of F‐MoO_3_ nanosheets prepared by vaporing the F‐MoO_3_ suspension on a copper mesh were displayed in **Figure** **2**a,b, which indicated that the size of F‐MoO_3_ was significantly decreased after ball milling, compared with the pristine α‐MoO_3_ (Figure S1, Supporting Information). The nanostructure of typical F‐MoO_3_ nanosheets was shown in a high‐magnification TEM image (Figure 2c). A MoO_3_ nanosheet with a length of ≈60 nm was densely packed by few‐layer nanosheets. The lattice fringes were confirmed in Figure 2d, which exhibited constant *d*‐spacings of 0.37 and 0.39 nm, corresponding to (200) and (002) planes, respectively. The inset of Figure 2d was the selective area electron diffraction (SAED) pattern of F‐MoO_3_ nanosheets, which confirmed the crystal structure of the as‐prepared F‐MoO_3_ nanosheets. The lateral size distribution was shown in Figure 2e. There were 150 pieces of MoO_3_ nanosheets counted in total. Meanwhile, the lateral size of MoO_3_ nanosheets is distributed from 20 to 200 nm. The thickness profile was further studied by atomic force microscopy (AFM). As shown in Figure 2f, the thickness of the as‐prepared F‐MoO_3_ nanosheets was 8–9 nm.

**Figure 3:**
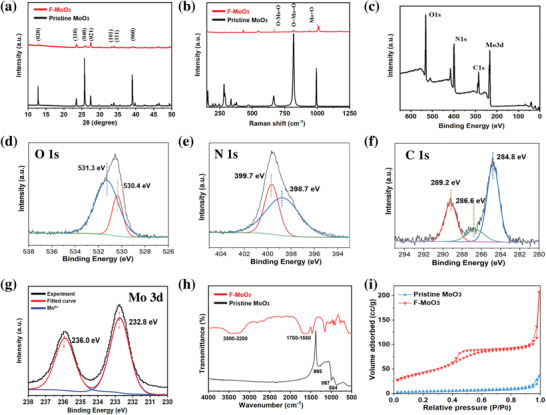
Spectroscopic characterizations. a) XRD patterns, b) Raman spectra, High‐resolution XPS spectra of F‐MoO_3_ on c) full scan, d) O 1s, e) N 1s, f) C 1s, g) Mo 3d, h) FTIR spectrum, and i) Nitrogen adsorption‐desorption isotherms of pristine MoO_3_ and F‐MoO_3_.

The crystal structure of F‐MoO_3_ was confirmed by X‐ray powder diffraction (XRD) (Panalytical X'Pert PRO diffraction system with a Cu Ka radiation) As shown in **Figure** **3**a, all the diffraction peaks are indexed to the orthorhombic crystal structure of MoO_3_ (a = 3.9450 Å, b = 13.8250 Å, c = 3.6940 Å).^[^ ^43^, ^44^ ^]^ The Raman spectra (Renishaw Raman spectrometer) of MoO_3_ powder were collected using a 532 nm laser and displayed in Figure 2b, which supported the crystal structure obtained from XRD. As shown in Figure 2b, the diffraction peaks at 665, 817, and 994 cm^−1^ were the asymmetric stretches and symmetric stretch of the terminal oxygen atom (Mo^6+^ = O), respectively, relating to the MoO_3_ orthorhombic crystal phase.^[^ ^45^ ^]^ Meanwhile, the high intensity with sharp background peaks suggests highly ordered MoO_3_ nanostructures.^[^ ^46^ ^]^ Besides, Raman spectroscopy was employed to determine the chemical properties of the F‐MoO_3_ (Figure S2, Supporting Information). Several diffraction peaks at 283, 431, 1183, 1648, and 2760 cm^−1^, were assigned to the bending of terminal oxygen, carbonyl (C=O) group, NH, and CONH, respectively.^[^ ^47^, ^48^, ^49^, ^50^ ^]^

The chemical component of F‐MoO_3_ nanosheets was confirmed by X‐ray photoelectron spectroscopy (XPS) (ESCALab MKII X‐ray photoelectron spectrometer) measurements. As shown in Figure 3c, the XPS survey spectrum revealed the existence of molybdenum (Mo), oxygen (O), carbon (C), and nitrogen (N) of which peaks at 231.42, 284.28, 398.28, and 530.1 eV corresponds to Mo 3d, C 1s, N 1s, O 1s signals, respectively. The high‐resolution XPS spectra were used to further study the binding energy of surface functional groups (Figure 3d–f). The high‐resolution XPS spectrum of O 1s (Figure 3d) presented two peaks at 531.3 and 530.4 eV, which can be deconvoluted into C=O bonds and Mo—O bonds, respectively.^[^ ^51^, ^52^ ^]^ It is necessary to notice that there is a little shift in these peaks compared with current reports due to the oxygen vacancy.^[^ ^52^ ^]^ Figure 3e displays the N 1s spectrum, which can be deconvoluted into two peaks with binding energy at 398.7 and 399.7 eV, connecting to C—N bindings and N—H bindings, respectively.^[^ ^53^, ^54^ ^]^ According to Figure 3f, the XPS spectrum of C 1s can be deconvoluted into three peaks. The peak at 284.8 eV was related to C—H binding, while the peaks at 286.6 and 289.2 eV were attributed to C—N bindings and C=O bindings, respectively.^[^ ^55^, ^56^ ^]^ The results were in good agreement with the Raman spectrum, demonstrating the existence of CONH and NH_2_ groups. Furthermore, the high‐resolution Mo 3d spectrum (Figure 3g) can be distinguished as two major contributor peaks located at 232.9 and 236.1 eV, which were typical characteristic peaks of the 3d doublet of Mo^6+^.^[^ ^57^, ^58^ ^]^

The functional group continued to be studied by the Fourier transform infrared (FTIR) spectrum. (Nicolet 7199 FT‐IR) According to Figure 3h, the pristine MoO_3_ exhibited the characteristic peaks at 995, 867, and 584 cm^−1^, while the FTIR spectrum of as‐prepared F‐MoO_3_ nanosheets showed different peaks. The shoulder extended from 3200 to 3500 cm^−1^ can be ascribed to —OH stretching vibrations and —NH_x(x=1,2)_ stretching vibrations. The peaks from 1550 to 1700 cm^−1^ were assigned to stretching vibrations of the attached CONH groups (C=O and linkage of CONH groups).^[^ ^59^ ^]^ The peaks from 500 to 880 cm^−1^ related to the Mo—O—Mo stretching vibration, and the range from 950 to 1000 cm^−1^ were assigned to the characteristic bonds of M=O.^[^ ^60^ ^]^ Furthermore, Three peak shifts of F‐MoO_3_ can be observed at 945, 1049, and 1153 cm^−1^, which suggests partly Mo=O bonds broken and attached by functional groups.^[^ ^57^ ^]^ It is in agreement with the results as indicated in the above SAED, Raman, and XPS data. Therefore, the existence of functional groups, including NH_x__(x=1,2)_, CONH, and OH groups, was confirmed. They were attached to the surface of the F‐MoO_3_ nanosheets and facilitated F‐MoO_3_ nanosheets to form a stable homogeneous suspension over 2 weeks. (Figure S3, Supporting Information).

The specific surface area data of pristine and F‐MoO3 was confirmed by Brunauer–Emmett–Teller (BET) (Quantachrome Autosorb iQ‐MP/XR), shown in Figure 3i. It suggested that the specific surface area of F‐MoO_3_ was 106 cc g^−1^ (Barrett‐Joyner‐Halenda model) or 161.194 cc g^−1^ (BET model), which was much higher than the pristine MoO_3_ (5 cc g^−1^). The results indicated that the pristine MoO_3_ was thoroughly exfoliated into nanosheets after the high‐energy ball milling process.

### 2.2 The Performance of Dye Removal from Water

**Figure 4:**
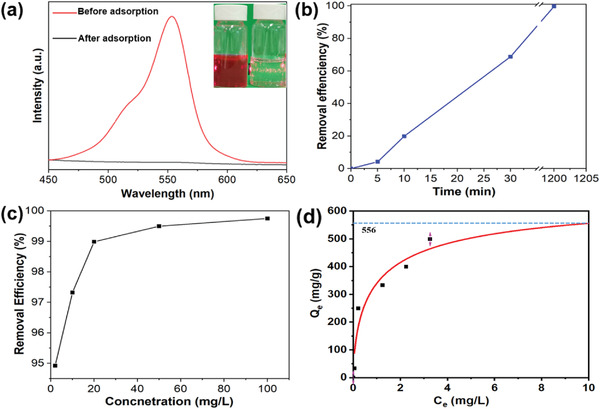
a) UV–vis absorbance plots for RhB aqueous solution in presence of MoO_3_ nanosheets over time. The inset showed the RhB solution before (left) and after adsorption (right). b) The removal efficiency of MoO_3_ nanosheets in 100 mg L^−1^ RhB solutions. c) The effect of adsorbate dosage on RhB removal. d) Absorption isotherm of RhB on MoO_3_ nanosheets.

The ultraviolet‐visible spectra (UV‐vis)(Cary 300) of the RhB solution before and after adsorption was shown in **Figure** **4**a. The RhB has a characteristic peak at 554 nm, but the intensity of the peak vanished after the adsorption, implying the RhB molecules were removed after adsorption by F‐MoO_3_ nanosheets. The inset showed a significant color disappearance after adsorption, supporting the removal of the RhB molecules from the aqueous solution. The effect of contact time is also an important factor in dye adsorbing process. In Figure 4b, the effect of contact time on the adsorption of F‐MoO_3_ nanosheets was presented. Approximately 70% of RhB molecules were removed within 30 min, suggesting high efficiency for RhB removal. Besides, the effect of adsorbent dosage was another important factor affecting the adsorption capacities. As shown in Figure 4c, the removal quantity of RhB increased with the decrease of absorbate dosage, due to higher absorbent dosage inducing more active binding sites. The adsorption isotherm (Figure 4d) fitted by the Langmuir model exhibits a maximum adsorption capacity Q_m_ of 556 mg g^−1^ (RhB concentration is 100 mg L^−1^), which was significantly higher than the relevant previous reported materials, listed in **Table** **1**. The results revealed that the F‐MoO_3_ nanosheets can be a promising candidate for RhB removal from an aqueous solution.

**Table 1:** The maximum RhB adsorption capacity of different adsorbents

Adsorbent	Q_m_ [mg g^−1^]	Reference
MoO_2_ – nanoparticles	256	[59]
MoO_3_ nanowires	321	[64]
MoO_3_ – hierarchical	204	[65]
MoO_3_ nanorod	326.8	[66]
MoO_3_ nanosheets	556	This work

The effect of solution pH and ionic strength on the adsorption of RhB was investigated under acidic condition at room temperature. The initial pH of RhB (100 mg L^−1^) is ≈3.7, and then a certain amount of hydrochloric acids (H^+^) or sodium hydroxides (OH^−^) was respectively added into the solution for adjusting the pH. As shown in Figure S5 (Supporting Information), a trend was clearly observed that the adsorption capacity increased with increasing the solution pH, corresponding to the protonation degree of functional groups on F‐MoO_3_ nanosheets. Furthermore, the adsorbed RhB on the F‐MoO_3_ nanosheets can be quickly desorbed by dispersing in ethanol. Figure S6 (Supporting Information) showed the recycling ability of RhB onto F‐MoO_3_ nanosheets. The recovery rates of the RhB solution on F‐MoO_3_ nanosheets reached 99.41% after desorption by dispersing in ethanol, even though the adsorption capacity slightly decreased from 477 to 474 mg g^−1^, indicating an excellent recovery ability. Moreover, tap water (containing various cationic ions) was used to prepare an RhB solution (100 mg L^−1^) for stimulating the real water. As shown in Figure S7 (Supporting Information), the adsorption capacity of RhB on F‐MoO_3_ nanosheets slightly decreased from 477 to 416 mg g^−1^ when other cationic ions existed. The results indicate that the competitive adsorption between the other cationic ions existing in tap water and RhB on F‐MoO_3_ nanosheets led to the reduction of the adsorption capacity.^[^ ^61^ ^]^ Although the adsorption performance decreased in the stimulated real water, the adsorption capacity (416 mg g^−1^, RhB/F‐MoO_3_ nanosheets) is still much higher than other currently reported MoO_3_ materials (Table 1), showing great potential in industrial dye removal.

### 2.3 Adsorption Mechanism

**Figure 5:**
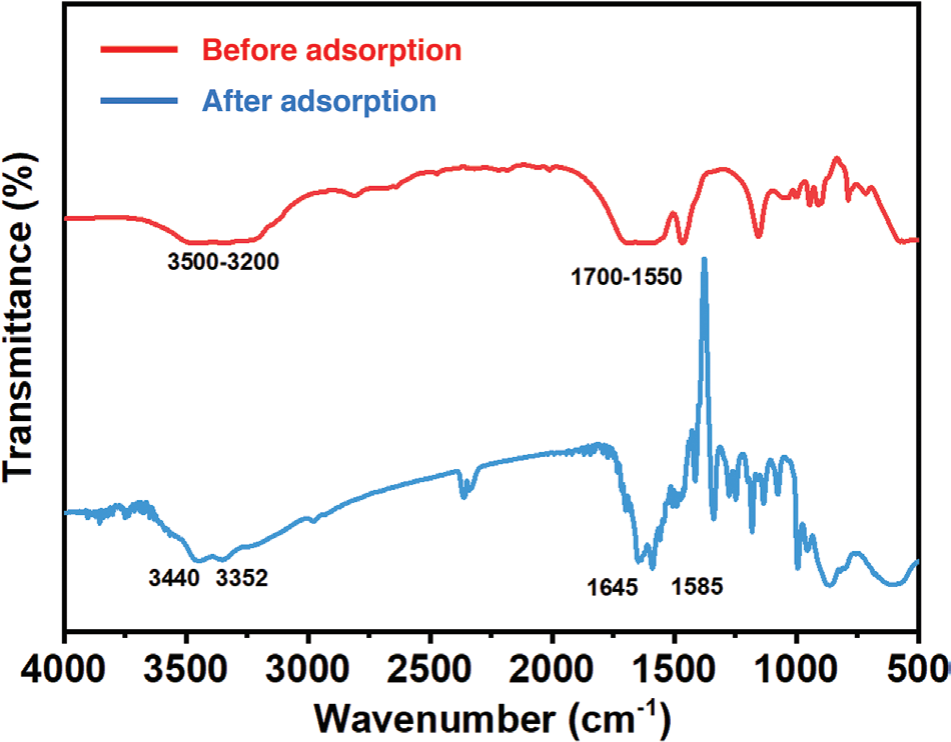
FTIR spectra of F‐MoO_3_ nanosheets before and after RhB adsorption.

To further investigate the adsorption mechanism, the FTIR of F‐MoO_3_ nanosheets before and after dye adsorption were performed to determine the interaction between cationic dyes and MoO_3_. As literature reported, several functional groups, including the CONH groups and OH groups, can affect the adsorption capacity of cationic dyes, while the NH groups will slightly decrease the adsorption capacity, corresponding to the sorption results obtained from the FTIR spectra (**Figure** **5**).^[^ ^62^ ^]^ The change in the intensity of characteristic peaks before and after RhB adsorption confirmed that the RhB molecules were attaching to the surface of F‐MoO_3_ nanosheets through the electrostatic interaction and hydrogen bonds. As shown in Figure 5, the peaks of the —NH_x(x=1,2)_ at 3440 and 3352 cm^−1^, C=O bond at 1645 cm^−1,^ and the linkage of the CONH groups (C—N, N—H) at 1585 cm^−1^ remained after RhB adsorption, indicating that the RhB molecules were not attached to the —NH_x(x=1,2)_ and —CONH groups. Meanwhile, the peak of —OH stretching vibrations (shoulder from 3257 to 3599 cm^−1^) vanished after RhB adsorption, demonstrating the functional groups occupied by the RhB molecules. Thus, by introducing new functional groups was not only increasing the adsorption capacities but also facilitatind the dispersal ability of F‐MoO_3_ in the aqueous solution. Moreover, the increasing negative charge in the aqueous solution was also a factor for high adsorption capacities. As shown in Tables S1 and S2 (Supporting Information), both pristine MoO_3_ and as‐prepared F‐MoO_3_ nanosheets exhibited negative potential values, −31.5 ± 0.6 and −45.5 ± 1 mV, respectively. The absolute ζ‐potential of F‐MoO_3_ nanosheets was increased after ball milling, which enhanced the strength of electrostatic interaction. Furthermore, the protonated functional groups enhanced the absolute ζ‐potential of the F‐MoO_3_ nanosheets by increasing the pH of the solution, leading to a higher adsorption capacity of RhB on F‐MoO_3_ nanosheets via electrostatic forces. Meanwhile, the adsorption capacity of RhB on F‐MoO_3_ nanosheets decreased in real water, due to the negatively charged active binding sites being partly occupied by the other cationic ions in tap water.^[^ ^61^ ^]^ Besides, the anionic dye (reactive black 5) was tested to verify if the RhB dye was adsorbed on F‐MoO_3_ nanosheets via electrostatic attractions. As shown in Figure S8 (Supporting Information), the concentration of the solution barely decreased after the adsorption, demonstrating that the negatively charged F‐MoO_3_ nanosheets can hardly adsorb anionic dyes. Therefore, it is concluded that the electrostatic interaction between RhB and functional groups on F‐MoO_3_ nanosheets mainly contributes to the high adsorption capacity of RhB on F‐MoO_3_ nanosheets. Meanwhile, it was reported that the surface structure and the specific surface area can affect the dye adsorption capacity as well.^[^ ^63^ ^]^ The increase of surface area on F‐MoO_3_ nanosheets (106 cc g^−1^) was another reason for the high adsorption capacities.

## 3 Conclusion

In this study, high‐energy ball milling method was successfully applied to produce F‐MoO_3_ nanosheets with the assistance of urea as the milling agent and functional group source by appropriately controlling the process parameters. According to the Raman, FTIR, and XPS analysis, functional groups (—OH, —CONH, —NH_x_ (x = 1, 2)) were recognized by attaching to the surface of MoO_3_ nanosheets. Then, the as‐prepared F‐MoO_3_ nanosheets were utilized to adsorb RhB from aqueous solutions. The obtained F‐MoO_3_ nanosheets as an adsorbent exhibited high adsorption capacity for RhB with 556 mg g^−1^, compared with other recently reported nanostructured MoO_3_. Several factors, including electrostatic interaction, surface complexation, and hydrogen bonding can affect the adsorption of RhB on F‐MoO_3_ nanosheets, while the electrostatic contact between surface functional groups and RhB molecules played a main role during the adsorption process. Thus, all features as reported demonstrated that the F‐MoO_3_ is a promising candidate for wastewater treatment.

## 4 Experimental Section

### Synthesis Method

F‐MoO_3_ nanosheets were prepared by modifying Lei's ball milling method.^[^ ^67^ ^]^ 10.5 g of commercial α‐MoO_3_ (Sigma, Molybdenum (VI) oxide, 99.5%) and urea (Sigma, ACS Reagent, 99.0–100.5%) mixtures with a 1:20 mass ratio and ten stainless steel balls (diameter = 10 mm) were placed in a stainless steel milling jar. Then, the mixtures were milled for 40 h at a speed of 500 rpm while jars were settled in the ball mill machine (Fritsch P7). After several times centrifuging with ethanol for 30 min at a speed of 12 000 rpm to remove urea, few‐layer F‐MoO_3_ nanosheets suspension and bulk MoO_3_ powder could be separated by centrifuging for 10 min at a speed of 3000 rpm. After freezing by liquid nitrogen for 30 min, the as‐prepared suspension was placed in the freeze‐dryer (Christ Beta 2–8 LSCbasic) for 48 h to obtain F‐MoO_3_ nanosheets powder.

### Adsorption Experiment

RhB was adopted to evaluate the dye removal abilities of the F‐MoO_3_ nanosheets. Dye solutions (RhB) with different concentrations were prepared by diluting a certain concentration dye solution (100 mg L^−1^) determined by UV spectra (554 nm for RhB). The calibration curve was fitted from the spectra of the standard solutions (*R* ^2^ > 0.99).

In a typical F‐MoO_3_ nanosheets absorption experiment, a certain amount of the F‐MoO_3_ nanosheets were added to 10 mL of RhB aqueous solution (100 mg L^−1^) in the dark condition under magnetic stirring for variation time (5–1200 min). After absorption, the suspension was centrifuged to remove F‐MoO_3_, and the amount of dye left in the supernatant was assessed by a UV spectrometer. The adsorption isotherm was obtained by adjusting the initial dye solution concentration. The removal efficiency (%Removal) and the equilibrium adsorption capacity (*Q*_e_, mg g^−1^) were calculated by the following Equations^[^ ^68^ ^]^

(1) $$\begin{array}{c} \%\text{Removal}=\frac{C_{o}-C_{e}}{C_{o}}\times 00\% \end{array}$$

(2) $$\begin{array}{c} Q_{e}=\frac{\left(C_{o}-C_{e}\right)V}{m} \end{array}$$

where *C*_o_ (mg L^−1^) and *C*_e_ (mg L^−1^) are the concentration of before and after absorption, respectively. The *m* (g) is the weight of the absorbent, and *V* (L) is the liquid volume.

## Conflict of Interest

The authors declare no conflict of interest.

## Supporting information

Supporting Information

## Data Availability

The data that support the findings of this study are available from the corresponding author upon reasonable request.
