# Supplementary material for: Functionalized MoO3 Nanosheets for High‐Efficiency RhB Removal
Source: Glob Chall. 2022 Dec 7;7(3):2200154. doi: 10.1002/gch2.202200154 (PMC10000286; doi:10.1002/gch2.202200154)
Supplement: Supplementary file 1 — Supporting Information [file GCH2-7-2200154-s001.pdf]

Global Challenges
Open Access

Supporting Information

for *Global Challenges,* DOI: 10.1002/gch2.202200154

Functionalized MoO_3 Nanosheets for High-Efficiency RhB Removal

Yuxi Ma, Lifeng Wang, Dan Liu, Yuchen Liu, Guoliang Yang, Yijun Qian, and Weiwei Lei**

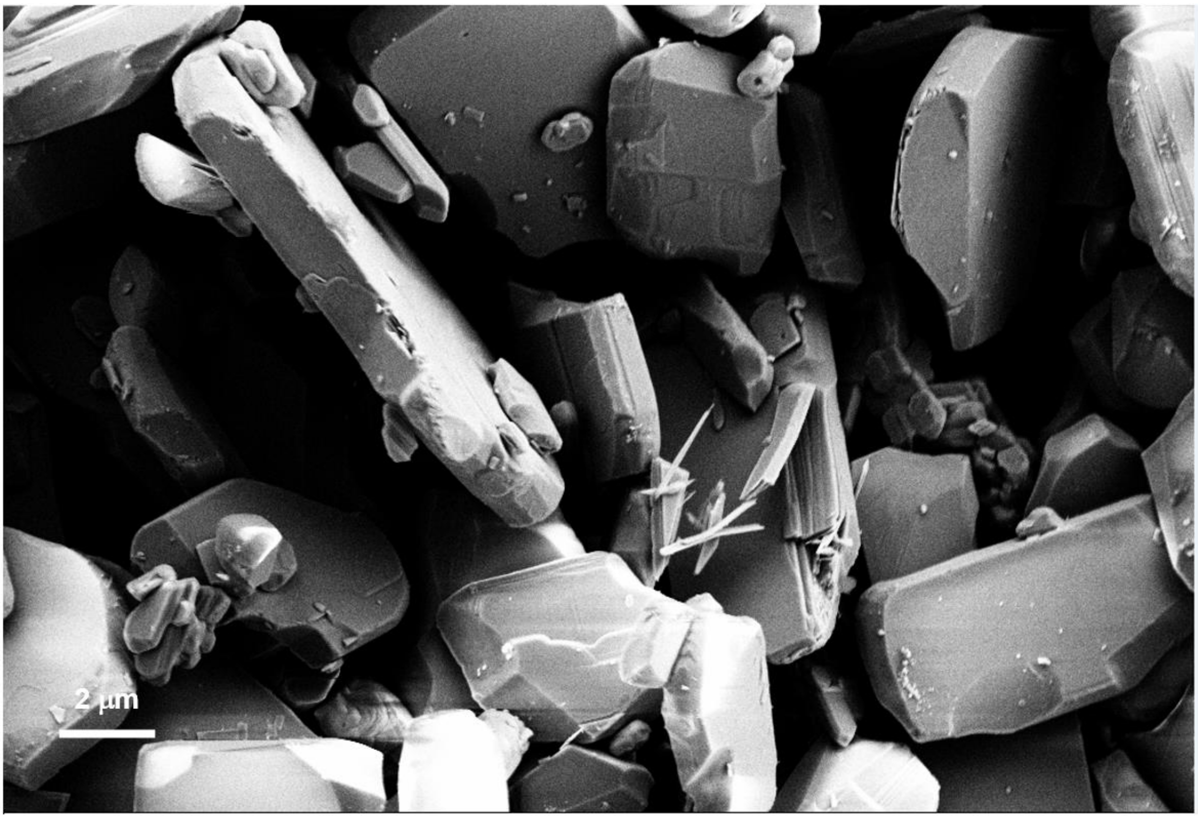

Figure S1. SEM image of pristine MoO_3.

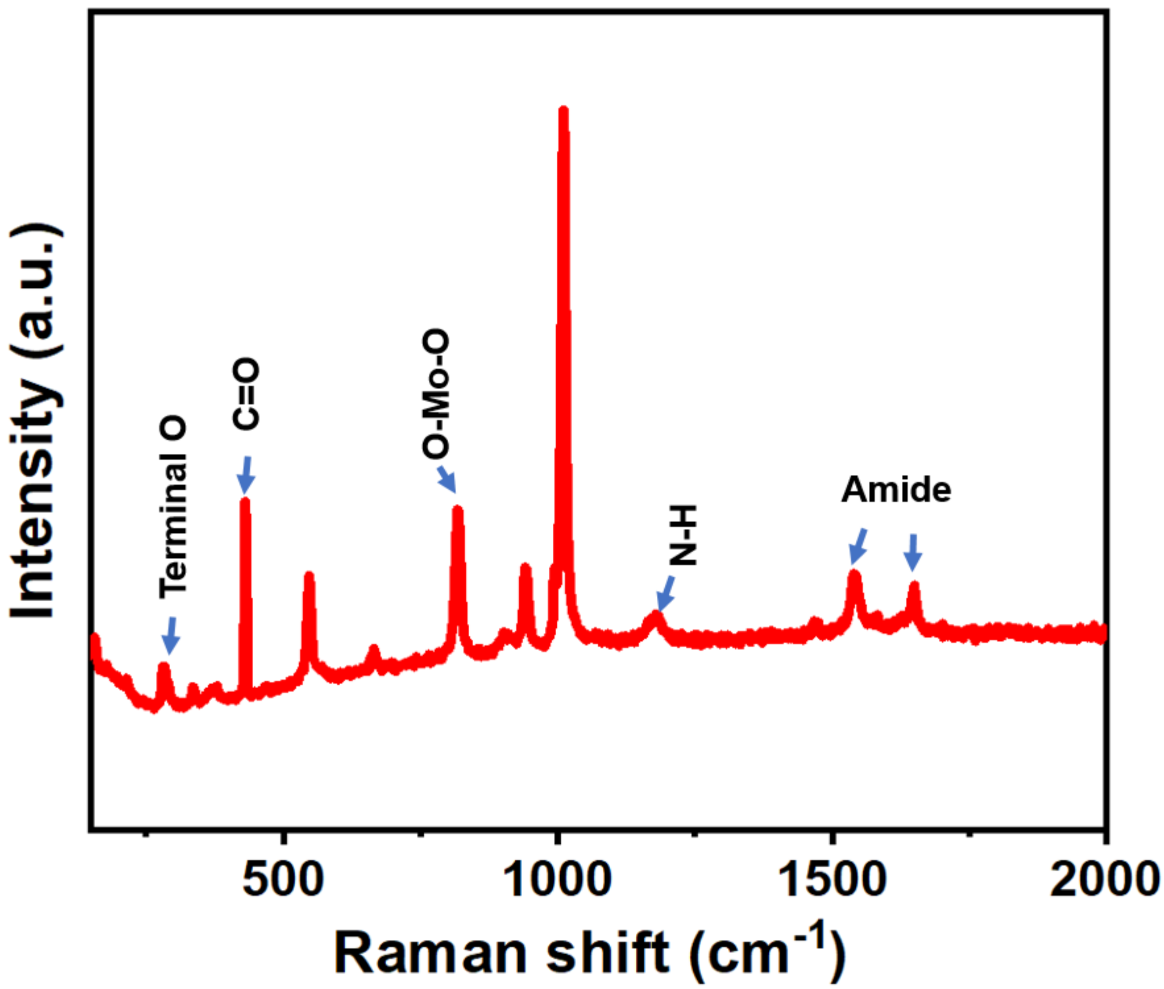

Figure S2. Full range Raman shift of F-MoO_3 nanosheets.

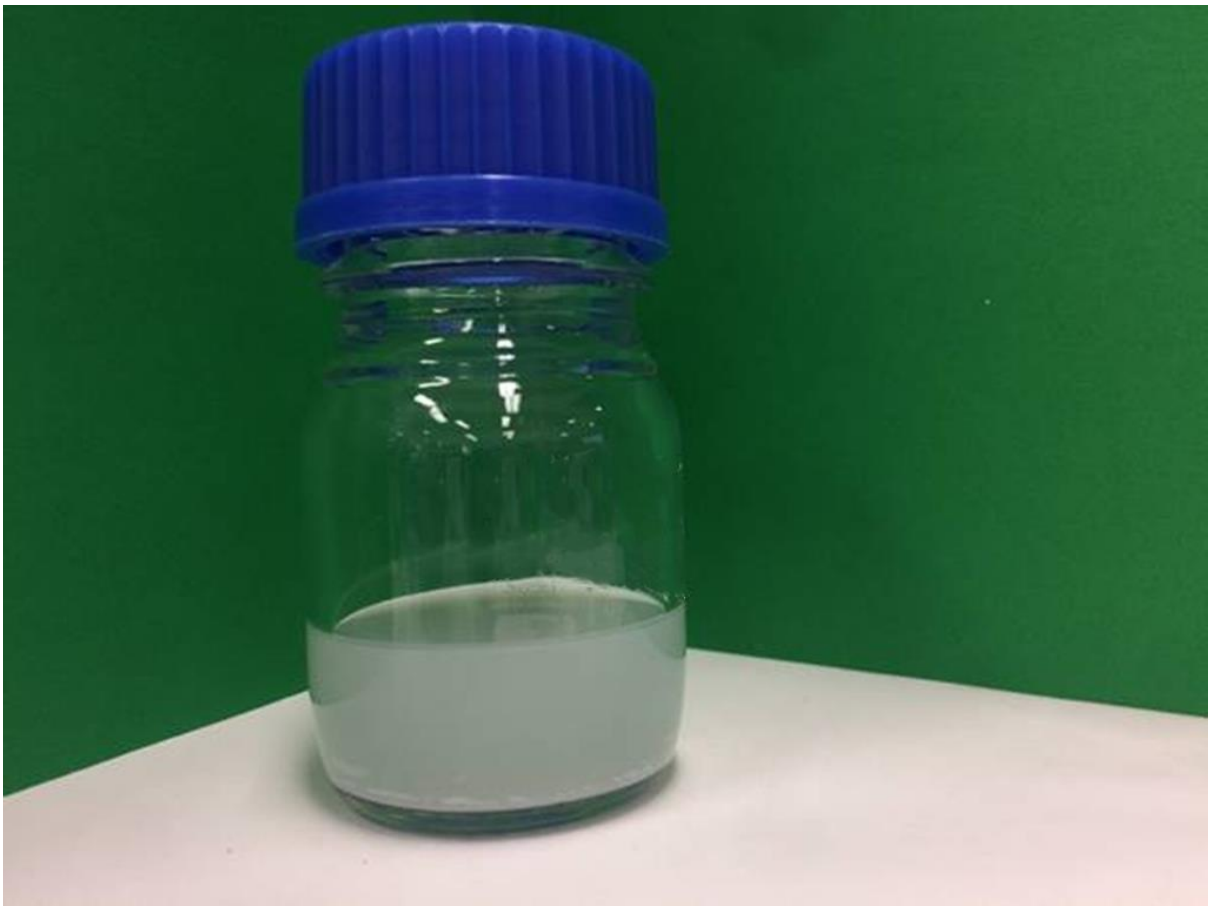

Figure S3. F-MoO_3 nanosheets aqueous dispersion for 2 weeks.

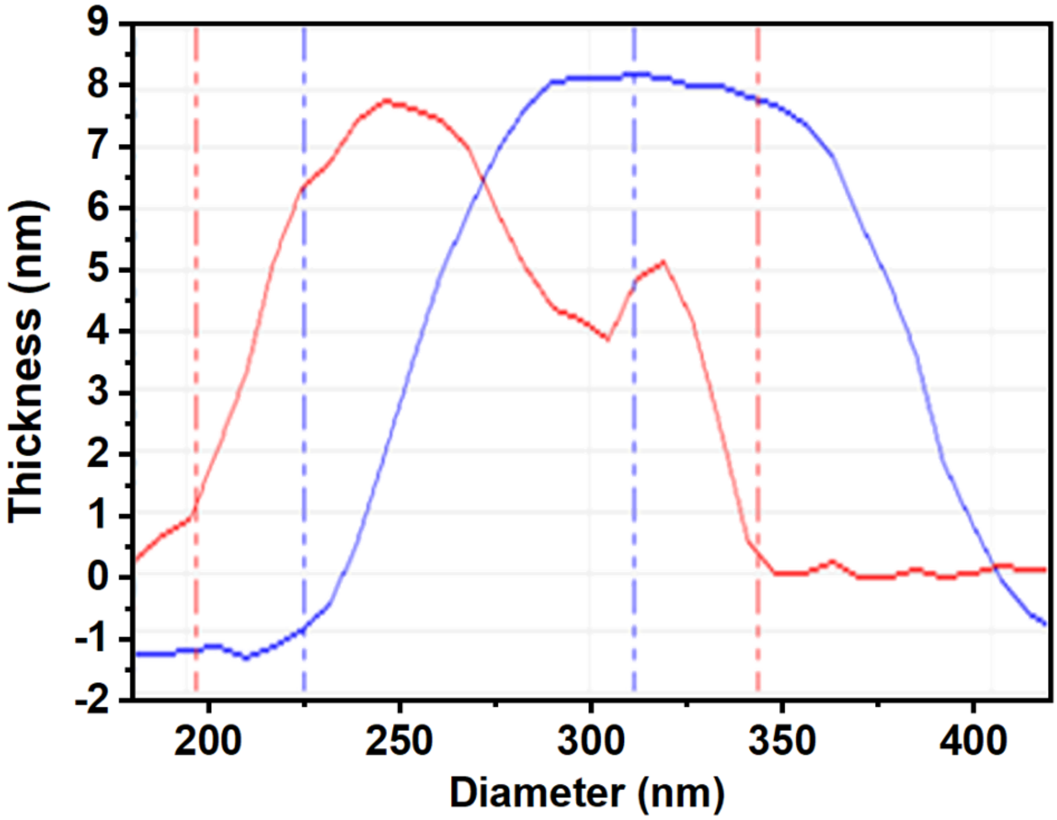

Figure S4. AFM profile of F-MoO_3

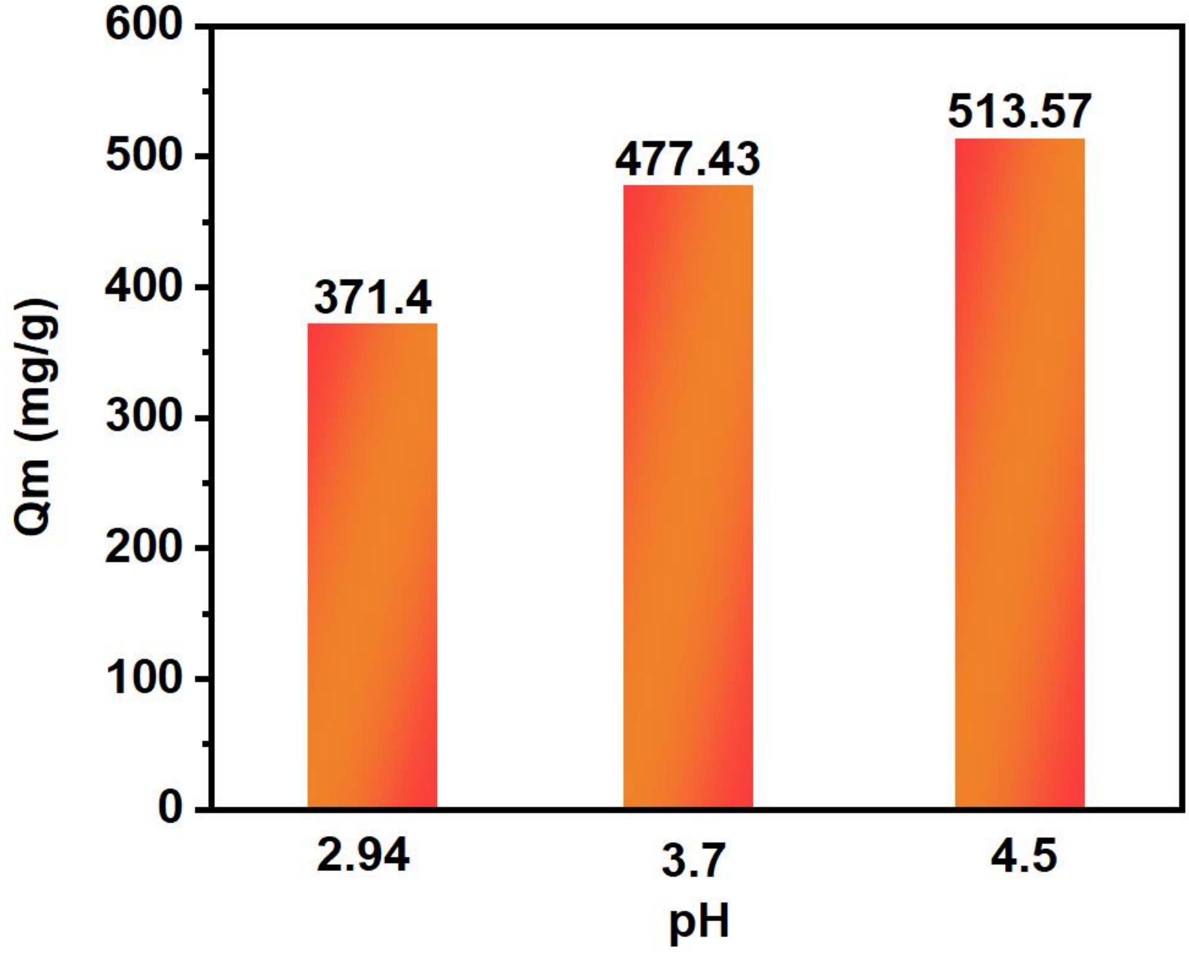

Figure S5. The effect of solution pH on the RhB removal.

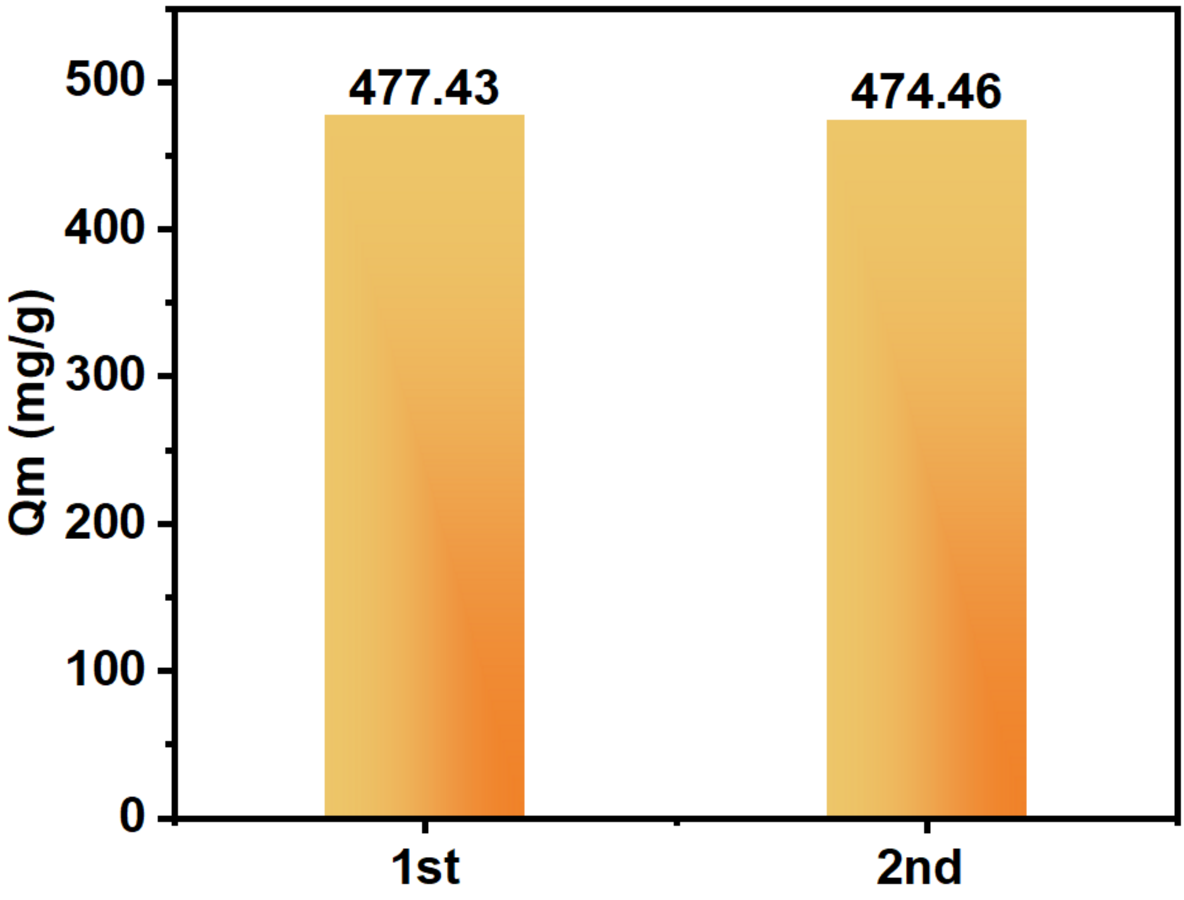

Figure S6 Recycle performance of F-MoO_3 on the RhB removal.

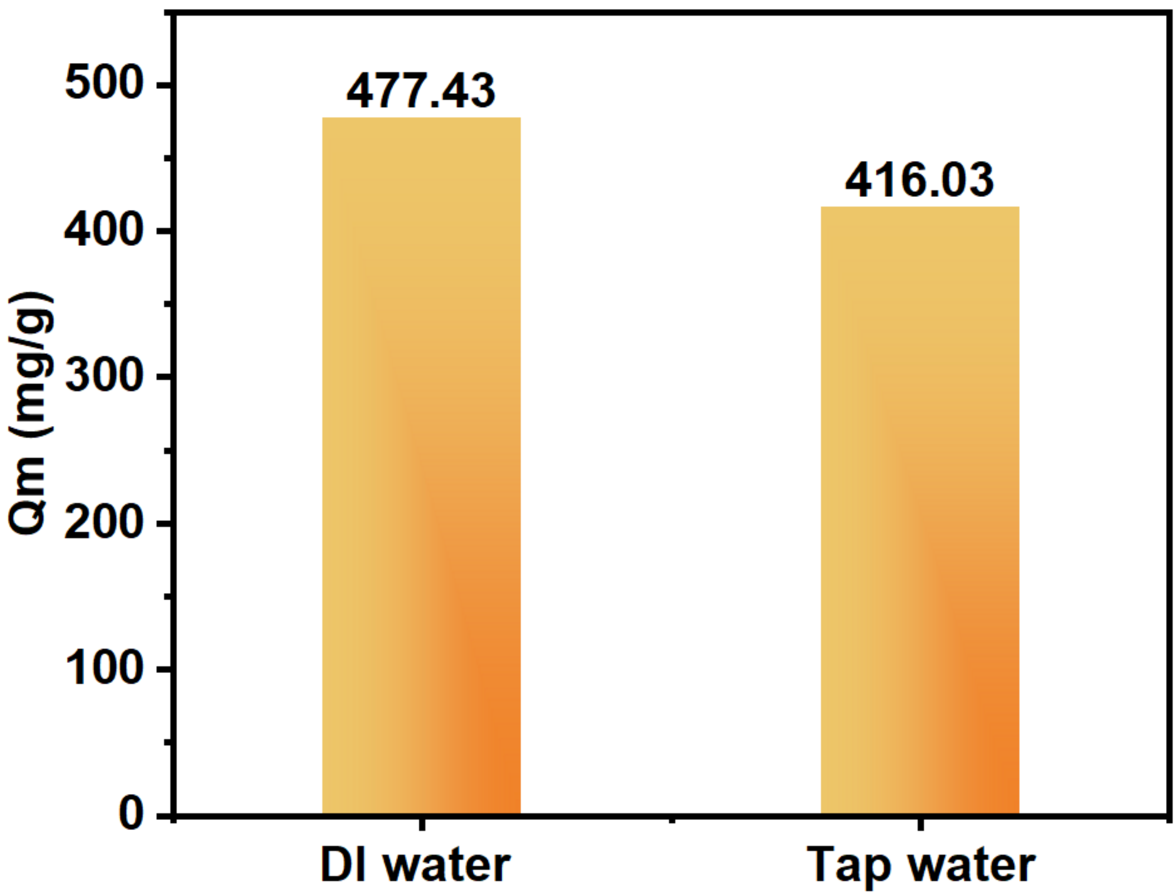

Figure S7. The effect of cationic ions on the RhB removal.

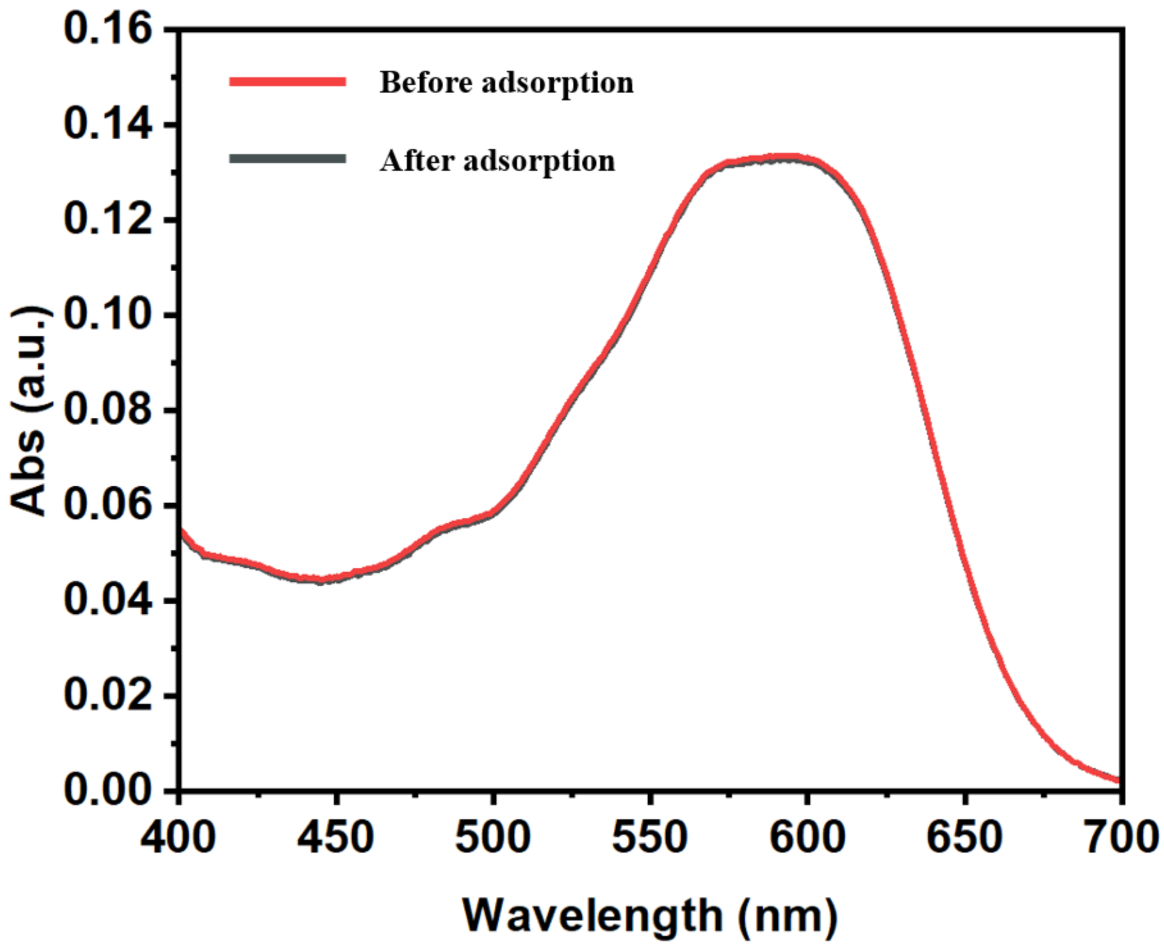

Figure S8. UV-vis absorbance plots for reactive black 5 aqueous solution in presence of MoO_3 nanosheets over time.

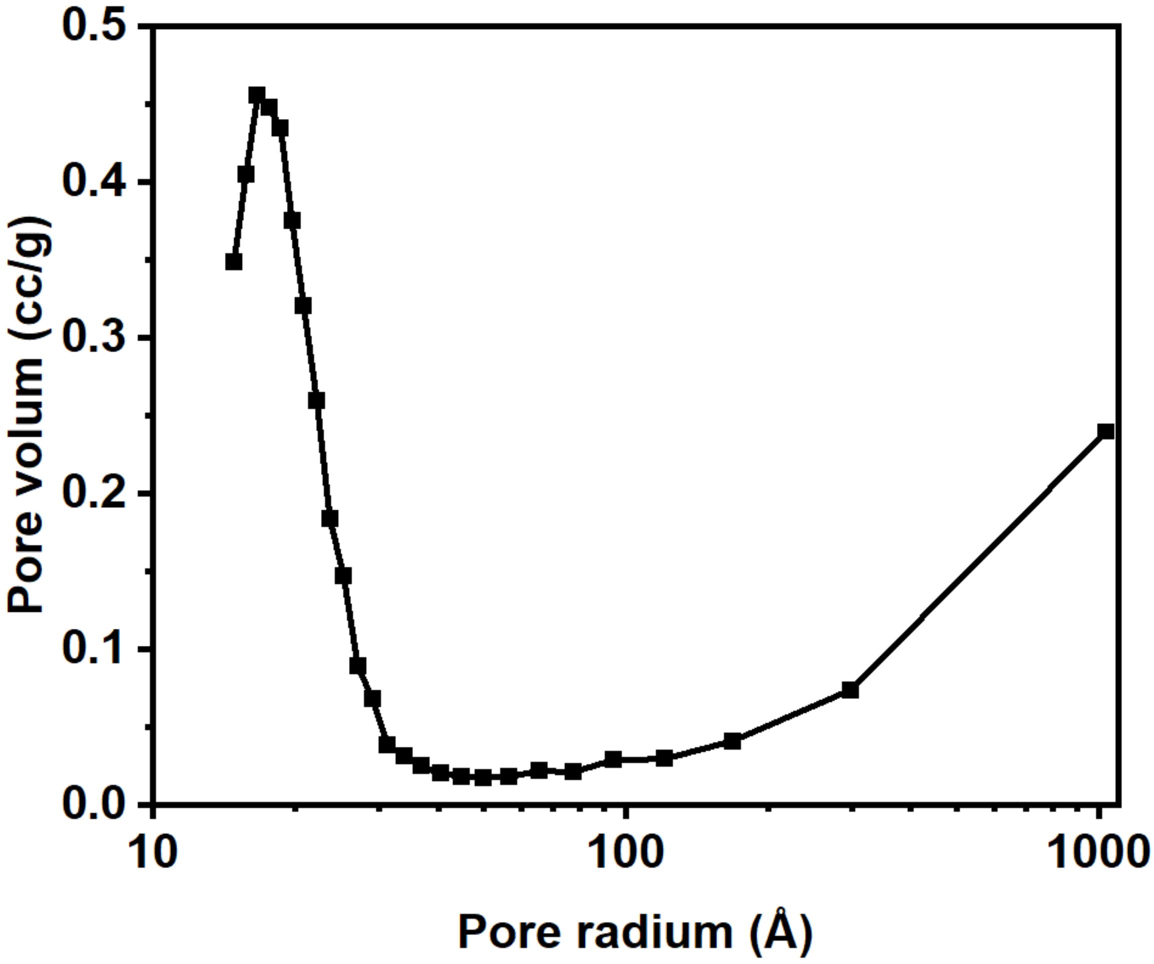

Figure S9. Pore size distribution plot of F-MoO_3.

Table S1. Zeta potential for pristine MoO_3.

T (°C)	ZP (mV)	Mob (μmcm/Vs)	Cond (mS/cm)
25	-31.4	-2.464	0.157
25	-30.9	-2.42	0.0578
25	-32.1	-2.517	0.0577

Table S2. Zeta potential for F-MoO_3.

T (°C)	ZP (mV)	Mob (µmcm/Vs)	Cond (mS/cm)
25	-45.1	-3.533	0.0559
25	-46.6	-3.654	0.0534
25	-44.6	-3.5	0.0545

Table S3. Distribution coefficient (Kd) value of the F-MoO3 nanosheets.

Q_e(mg g^{-1})	C_e (mg L^{-1})	K_d (L g^{-1})
33.81592	0.05191	651.4336
249.46686	0.21326	1169.7780
332.4529	1.24705	266.5915
399.01165	2.24709	177.5682
498.76475	3.26413	152.8017
